# Novel ESCC-related gene ZNF750 as potential Prognostic biomarker and inhibits Epithelial-Mesenchymal Transition through directly depressing SNAI1 promoter in ESCC

**DOI:** 10.7150/thno.38210

**Published:** 2020-01-01

**Authors:** Pengzhou Kong, Enwei Xu, Yanghui Bi, Xiaoqin Xu, Xue Liu, Bin Song, Ling Zhang, Caixia Cheng, Ting Yan, Yu Qian, Jian Yang, Yanchun Ma, Heyang Cui, Yuanfang Zhai, Binbin Zou, Xiangchen Liu, Yikun Cheng, Shiping Guo, Xiaolong Cheng, Yongping Cui

**Affiliations:** 1 Department of Pathology & Shanxi Key Laboratory of Carcinogenesis and Translational Research on Esophageal Cancer, Shanxi Medical University, Taiyuan, Shanxi 030001 P. R. China; 2 College of Letter & Science, University of California Berkeley, Berkeley, California, USA; 3 Department of Tumor Surgery, Shanxi Cancer Hospital, Taiyuan, Shanxi 030001, P.R. China

**Keywords:** ESCC, mutation, ZNF750, epithelial-to-mesenchymal transition, SNAI1.

## Abstract

**Background**: Cancer genomic studies have identified Zinc Finger Protein 750 (ZNF750) was a novel significantly mutated gene in esophageal squamous cell carcinoma (ESCC). This study was designed to determine the clinical value and molecular mechanisms of ZNF750 in the development of ESCC.

**Methods**: Genomic data from 4 reported ESCC cohorts were used to analyze the mutation profile of ZNF750. Tissue microarrays were used to detect its expression in 308 ESCC samples. Furtherly, the effects of ZNF750 on proliferation, colony formation, migration and invasion were tested in ESCC cells. PCR-array, chromatin immunoprecipitation (ChIP), luciferase reporter assays, and rescue assay were used to explore the mechanism of ZNF750. Correlation of ZNF750 with its target genes was analyzed in TCGA data from various SCC types.

**Results**: ZNF750 was frequently mutated in ESCC and the most common type was nonsense mutation. Its nucleus/cytoplasm ratio in ESCC was significantly lower than that in paired non-tumor tissues; it was an independent and potential predictor for survival in ESCC patients. Furtherly, ZNF750 knockdown significantly promoted proliferation, colony formation, migration and invasion in ESCC cells. PCR-array showed epithelial-to-mesenchymal transition (EMT) was the main biologic process affected by ZNF750. Moreover, ZNF750 directly bound to the promoter region of SNAI1 and depressed its activity. Decreased ZNF750 up-regulated SNAI1 expression and promoted EMT phenotype. SNAI1 knockdown partially reversed the malignant phenotype induced by ZNF750 knockdown. Further TCGA data analyses showed ZNF750 expression was positively correlated with E-cadherin and negatively correlated with SNAI1, N-cadherin and Vimentin in ESCC and other SCC samples.

**Conclusion**: Our results suggest that ZNF750 may act as a tumor suppressor by directly repressing SNAI1 and inhibiting EMT process in ESCC and other types of SCC.

## Introduction

Esophageal cancer is one of the most common malignant tumors of the digestive system, with high morbidity and mortality^1^. Despite the technical developments in diagnosis and treatment, this disease still tends to have a poor prognosis^2^, ^3^ and the 5-year survival rate ranges from 15% to 25%^2^. Histological types of esophageal carcinoma include two main types: esophageal squamous cell carcinoma (ESCC) and esophageal adenocarcinoma (EAC)^2^. ESCC is the principal histological type in China, which has the highest incidence and mortality compared with other countries^4^.

In our previous genomic study of 104 ESCC cases, we identified that, Zinc Finger Protein 750 (ZNF750), a novel member of the zinc-finger transcription factor family, was one of the significantly mutated genes, which might give selective advantages to ESCC cells and play an important role in the progress of ESCC^5^. Meanwhile, ZNF750 was also reported as a significantly mutated gene in other genomic studies of ESCC^6^-^9^.

Human ZNF750 gene is located on 17q25.3 and encodes a protein with a highly conserved C2H2 zinc finger domain and a nuclear localization signal (NLS) in its C-terminal^10^. ZNF750 protein mainly exists in squamous epithelium, acts as a transcription factor involved in terminal differentiation of epithelium and plays a role of suppressor genes associated with proliferation and promoting the transcription of genes associated with differentiation^10^, ^11^. As reported, the frameshift mutation and the variation of the promoter region in ZNF750 gene may lead to lower protein expression and result in the occurrence of seborrheic dermatitis-like psoriasis^12^-^14^. ZNF750 has a relatively higher expression in esophagus than in other organs^15^. Recent studies showed that low ZNF750 expression was correlated with lymph node metastasis^16^ and poor prognosis in ESCC patients^16^, ^17^. However, its role and mechanism in cancer remain unclear, especially in ESCC. Here, we verified the tumor-suppressor role of ZNF750 in ESCC and elaborated on its possible mechanisms that ZNF750 directly bound to the promoter region of Snail Family Transcriptional Repressor 1 (SNAI1) and down-regulated the expression of SNAI1 to inhibit the epithelial-mesenchymal transition (EMT) of ESCC cells, in order to provide useful clues for clinical treatment of ESCC.

## Results

### ZNF750 is frequently mutated in ESCC

**Figure 1:**
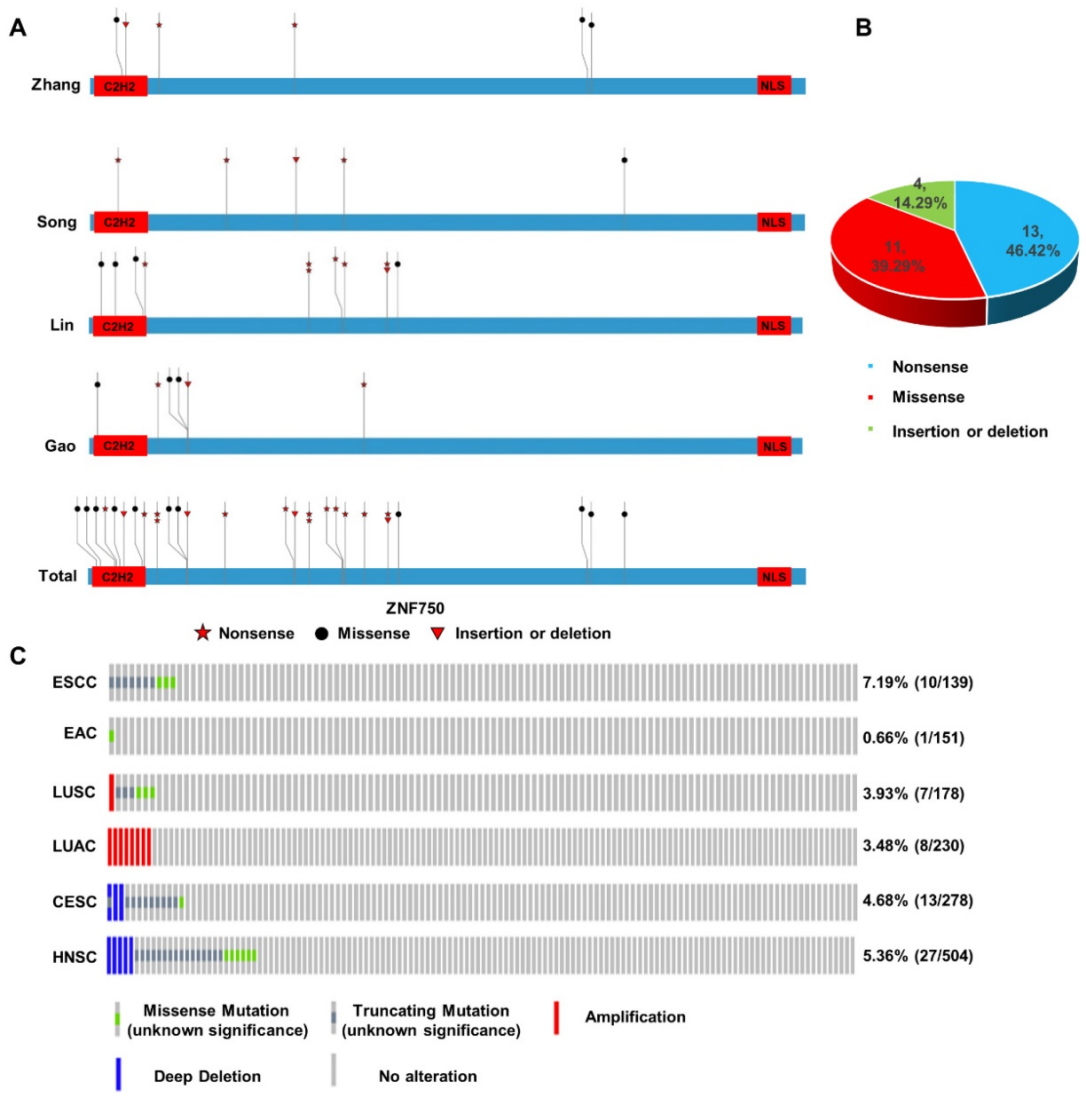
** Somatic mutation and copy number variation (CNV) profiles of ZNF750 in ESCC and other cancers. (A)** Somatic mutation of ZNF750 in 4 ESCC cohorts from China. **(B)** Proportion of different mutation types of ZNF750 in ESCC. **(C)** Difference of somatic mutation and CNV profiles in ESCC, EAC, LUSC, LUAC, HNSC and CESC through cBioPortal based on TCGA data.

In our previous work, we sequenced the genome of 104 ESCC tumors tissues and the matched adjacent non-tumor tissues from individuals recruited from the Taihang Mountains in North-central China and found ZNF750 was one of the significantly mutated genes in ESCC^5^. Here we integrated the genomic sequencing data from 4 reported ESCC cohorts from China^6^, ^7^, ^18^, including 424 tumor samples and matched non-tumor samples, which came from the Taihang Mountains and Chaoshan District of Guangdong Province, the two areas with high ESCC prevalence in China. We found ZNF750 was frequently mutated in all the four cohorts (Figure 1A). In Zhang's cohort^5^, the mutation rate is approximately 5.77% (6/104), including nonsense 2/6 (33.33%), missense 3/6 (50.00%) and insertion or deletion (indel) 1/6 (16.67%). In Song's cohort^7^, the mutation rate is nearly 5.68% (5/88), including nonsense 3/5 (60.00%), missense 1/5 (20.00%), indel 1/5 (20.00%). In Lin's cohort^6^, the mutation rate is about 9.24% (11/119), including nonsense 6/11 (54.54%), missense 4/11 (36.36%), insertion or deletion 1/11 (9.10%). And in Gao's cohort^18^, the mutation rate is closely 5.31% (6/113), including nonsense 2/6 (33.33%), missense 3/6 (50.00%), insertion or deletion 1/6 (16.67%). The total mutation rate is approximately 6.60% (28/424), including nonsense 13/28 (46.42%), missense 11/28 (39.29%), indel 4/28 (14.29%) in these four cohorts (Figure 1B). The most common type was nonsense mutation, which resulted in a premature stop codon and a truncated, incomplete and usually nonfunctional protein product, and lost its C-terminal NLS of ZNF750 and abrogated its nuclear localization. And the evolutionally conserved C2H2 DNA-binding domain is another hot region (8/28) mutated in ESCC.

The genetic data indicated that ZNF750 might act as a tumor suppressor gene in ESCC based on the rule of 20/20^19^. In addition, through cBioPortal based on the Cancer Genome Atlas (TCGA) or other cancer genome database, we found that the inactivating mutation rates of ZNF750 in different squamous cell carcinomas (SCC) were much higher than those in the corresponding adenocarcinomas (Figure 1C). In ESCC, the ratio of truncating and missense mutation was 7.19% (10/137); however, it was only 0.7% (1/151) in EAC. Similarly, the ratio of truncating and missense mutation was 3.93% (7/178) in lung squamous cell carcinoma (LUSC), however, there was no truncating or missense mutation of ZNF750 gene in lung adenocarcinoma (LUAC). In cervical squamous cell carcinoma (CESC) and head and neck squamous cell carcinoma (HNSC), the rates were 4.68% (13/278) and 5.36% (27/504) respectively. Therefore, the somatic mutation of ZNF750 may be a special driving event in the occurrence and development of ESCC, even in other types of SCC.

### ZNF750 nucleus/cytoplasm ratio is correlated with the prognosis of ESCC patients

**Figure 2:**
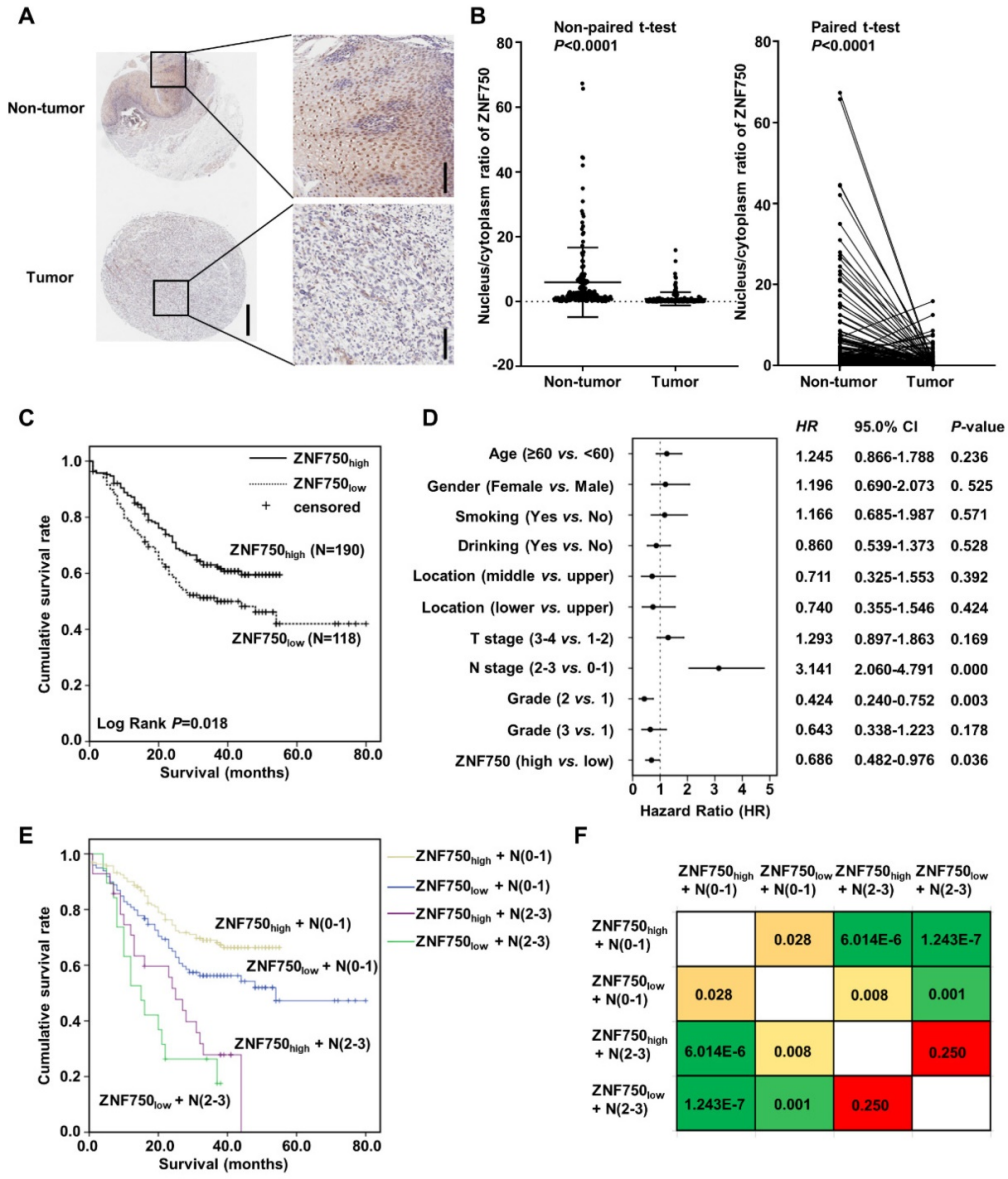
** Nucleus/cytoplasm ratio of ZNF750 is correlated with the prognosis of ESCC patients. (A)** Representative images of ZNF750 protein expression in tumor tissues and adjacent non‑tumor tissues from paraffin‑embedded formalin‑fixed ESCC tissue microarrays containing 308 tumors and corresponding non‑tumor tissues by IHC. ESCC tissues were stained by rabbit anti-ZNF750 antibody and counterstained by hematoxylin. ZNF750 was stained with brown and nuclei were stained with blue. Left bar=500 μm, right bar=100 μm. (**B**) Comparison of the nucleus/cytoplasm ratio of ZNF750 expression in paired ESCC tumor tissues and non‑tumor tissues using a non-paired t-test and paired t-test; *P* <0.001. **(C)** Kaplan-Meier survival plot showed the patients with ZNF750_high_ had better survival than those with ZNF750_low_. Log Rank *P* = 0.018. **(D)** Multivariate analysis showed the nucleus/cytoplasm ratio of ZNF750 was an independent predictive factor for overall survival in ESCC (HR = 0.686, 95 % CI: 0.482-0.976, *P* = 0.036). **(E)** Combination of ZNF750 and N stage can effectively divide the ESCC patients into four groups that have different survival rates. **(F)** The pairwise comparison matrix of the four groups divided by the combination of ZNF750 and N stage and the Log Rank *P* values were shown.

The genetic data indicated that ZNF750 might act as a tumor suppressor gene in ESCC. To detect the protein level of ZNF750 in ESCC, we detected its expression in several paired of fresh tumors with or without ZNF750 mutation. We found ZNF750 was significantly decreased in tumors compared to the matched adjacent non-tumor tissues but there was no significant difference between mutant and wild-type (Figure S1). We furtherly analyzed the protein level of ZNF750 in ESCC samples using tissue microarray (TMA), which included 308 ESCC and paired adjacent non‑tumor tissues. The results showed ZNF750 was mainly expressed in nucleus and cytoplasm of the non-tumor tissues and ESCC tissues (Figure 2A), in accord with the results of R. Otsuka 16 and our previous results 5. As a transcription factor, ZNF750 plays its role in the cell nucleus and loses its function in cytoplasm, and its nuclear translocation is important for its function. Consequently, we analyzed the nucleus/cytoplasm ratio of ZNF750 protein in clinical samples. The results showed the nucleus/cytoplasm ratio of ZNF750 in ESCC was significantly lower than that in paired non-tumor tissues (Figure 2B, *P* < 0.001). According to the nucleus/cytoplasm ratio of ZNF750, we divided the patients into two groups: patients with a lower nucleus/cytoplasm ratio (named as ZNF750_low_) and patients with a higher ratio (named as ZNF750_high_) (Figure S2). Then we analyzed the correlation between the nucleus/cytoplasm ratio of ZNF750 and the clinical variables in ESCC and evaluated its clinical value. The results showed the nucleus/cytoplasm ratio of ZNF750 was related to the invasion depth (T stage) (*P* = 0.061) and survival status (*P* = 0.024) of ESCC patients (Table 1). The patients with ZNF750_low_ had a deeper invasion and a worse prognosis compared with the ZNF750_high_ patients. Furtherly, Kaplan-Meier survival analysis showed the patients with ZNF750_low_ had a worse survival than those with ZNF750_high_ (Log Rank *P* = 0.018, Figure 2C). The multivariate analysis showed that N stage (Hazard Ratio (HR) = 3.141, 95 % CI: 2.060-4.791, *P* < 0.001) and the nucleus/cytoplasm ratio of ZNF750 were independent predictive factors for overall survival (HR = 0.686, 95 % CI: 0.482-0.976, *P* = 0.036) (Figure 2D). Furtherly the combination of ZNF750 and N stage could effectively divide the ESCC patients into four groups, which had different survival rates (Figure 2E-F, Table S1). The results indicated its decrease and location change might play important roles in the tumorigenesis and development of ESCC. Furthermore, ZNF750 was related with the survival status in the patients with age ≥ 60 (*P* = 0.041), male (*P* = 0.020), smoking (*P* = 0.033), drinking (Breslow *P* = 0.049), T stage =1+2 (*P* = 0.054), N stage = 0-1 (*P* = 0.028), TNM stage = Ⅲ+Ⅳ (*P* = 0.066), Grade = 1 (*P* = 0.010) (Figure S3 and S4).

### ZNF750 inhibits the ability of proliferation, colony formation, migration and invasion of ESCC cells in vivo and in vitro

**Figure 3:**
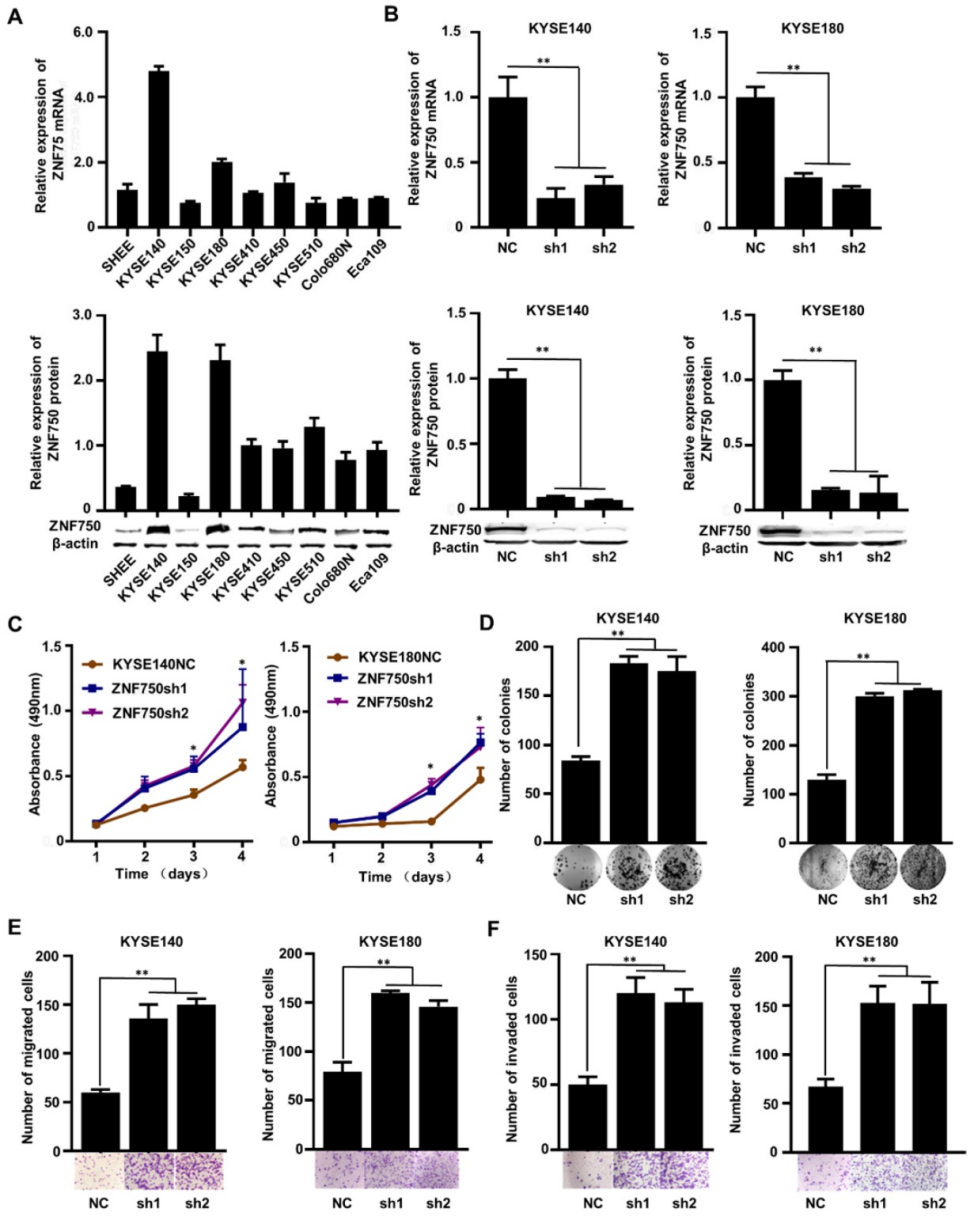
** The effect of ZNF750 knockdown on ESCC cell lines**. **(A)** ZNF750 mRNA and protein expression levels in 9 ESCC cell lines. **(B)** Endogenous ZNF750 was knocked down using shRNA carried by lentivirus. **(C)** ZNF750 knockdown promoted the proliferation ability of ESCC cells.** (D)** ZNF750 knockdown promoted the colony formation of ESCC cells. **(E)** ZNF750 knockdown promoted ESCC cell migration in KYSE140 and KYSE180 cells.** (F)** ZNF750 knockdown promoted ESCC cell invasion in KYSE140 and KYSE180 cells. (* *P* <0.05, ** *P* <0.01)

**Figure 4:**
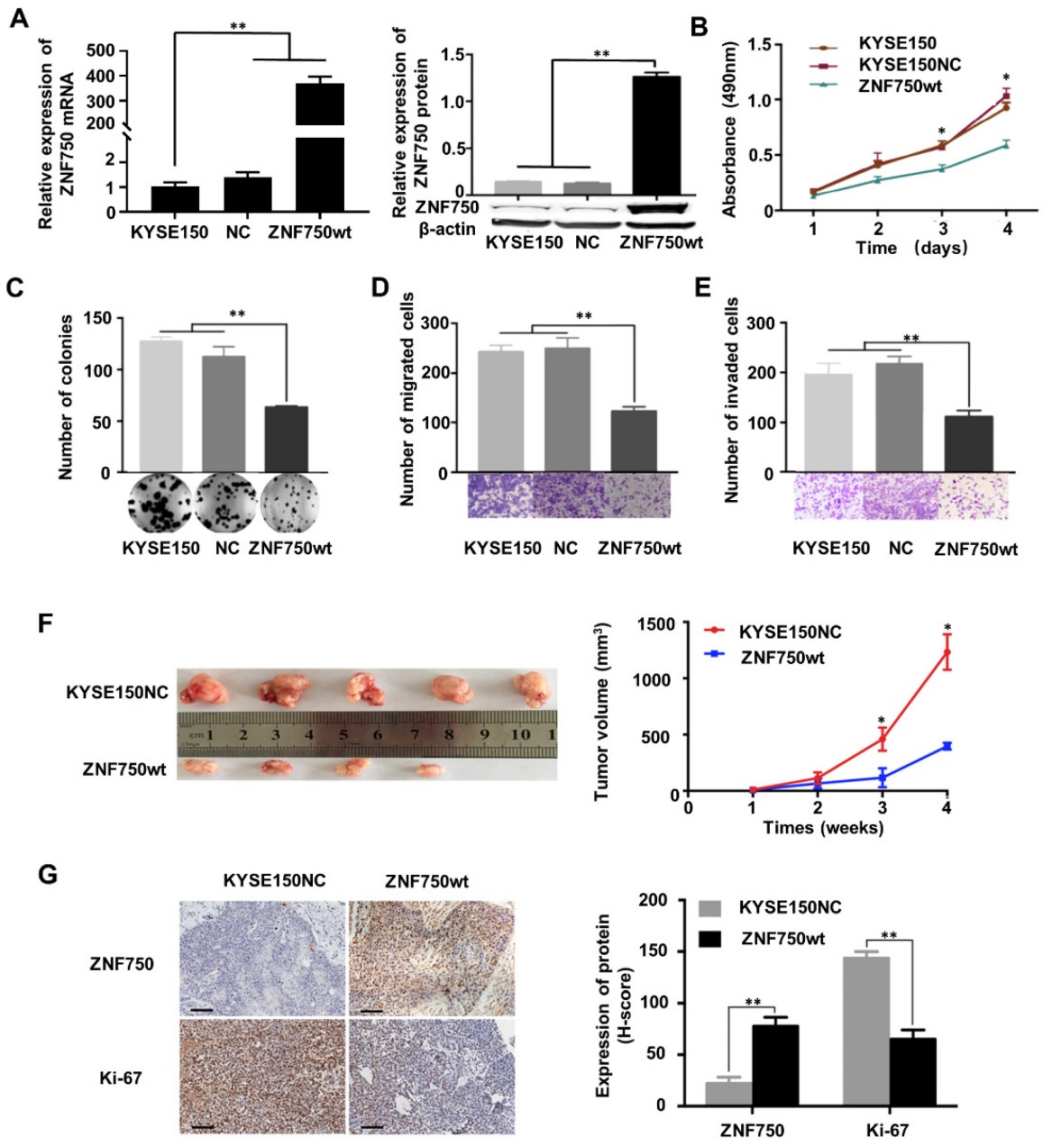
** ZNF750 overexpression significantly inhibited tumor growth in vitro and in vivo**. **(A)** ZNF750 overexpression in KYSE150 cells. **(B)** ZNF750 overexpression inhibited the proliferation ability of ESCC cells. **(C)** ZNF750 overexpression inhibited the colony formation of ESCC cells. **(D)** ZNF750 overexpression inhibited ESCC cell migration. **(E)** ZNF750 overexpression inhibited ESCC cell invasion. **(F)** ZNF750 overexpression significantly inhibited tumor growth in vivo. Left: ESCC tissues in ZNF750-overexpression group and the control group; Right: tumor growth curve. **(G)** IHC assay showed Ki-67 expression in ZNF750 overexpressed xenograft tumor tissue and the control group. Scale bar = 100μm. (* *P* <0.05, ** *P* <0.01)

The genetic data and the IHC analysis of ESCC samples indicated that ZNF750 might act as a tumor suppressor gene in ESCC. To verify the role of ZNF750 in ESCC, we measured ZNF750 expression levels in 9 ESCC cell lines by quantitative real-time PCR (RT-qPCR) and Western blot. The results showed that ZNF750 gene was relatively higher in KYSE180 and KYSE140 cells but lower in KYSE150 cells (Figure 3A). Thus, we inhibited the expression of ZNF750 in KYSE180 and KYSE140 cells using its short hairpin RNA (shRNA) by lentivirus (Figure 3B), then detected the changes in cell phenotypes, such as proliferation, invasion, migration and colony formation. The results showed that the abilities of cell proliferation, invasion, migration and colony formation were significantly increased when ZNF750 gene was down-regulated in KYSE140 cells and KYSE180 (Figure 3C-F). Then we transfected HA-tagged wild-type ZNF750 (ZNF750-wt) plasmid into KYSE140 cells and the immunofluorescence result showed ZNF750 was mainly located in the nucleus (Figure S5). Overexpression of ZNF750 gene in KYSE150 cell line (Figure 4A) significantly decreased cell proliferation, colony formation, invasion, and migration (Figure 4B-E), compared with the empty-vector group and parental cells.

To further confirm the tumor suppressor role of ZNF750 in ESCC, we establish a subcutaneous transplantation tumor model in BALB/c-nu mice using KYSE150 cells with wild-type ZNF750 stable overexpression (KYSE150-ZNF750wt) and the negative control cells (KYSE150-NC). After 4 weeks, mice were sacrificed and tumors were stripped. The results showed that tumor growth rate of ZNF750wt group was significantly slower than that of the NC group (t-test, *P* < 0.01, Figure 4F). The xenograft mice model results showed that the mean tumor volume of the control group, SCR group and the ZNF750wt group were (1212.41 ± 157.84) mm^3^, (394.74 ± 33.83) mm^3^, respectively.

We further immunohistochemically stained the tumor tissue samples with anti-ZNF750 and anti-Ki-67 antibody and found that Ki-67 staining intensity in ZNF750 overexpressed group (ZNF750wt) was significantly weaker than that of the control group (KYSE150NC) (Figure 4G left). The results showed that the H-score of Ki-67 in ZNF750wt group (144.62 ± 6.15) was significantly lower than that in the control group (64.62 ± 8.01) (t-test, *P* < 0.001, Figure 4G right). Taken together, the results suggested that ZNF750 gene may act as a tumor suppressor in ESCC and its inactivating-mutation or decreased expression may promote the malignant phenotype of ESCC cells, such as invasion, migration and so on.

### ZNF750 inhibits the EMT process in ESCC

To explore the potential molecular mechanism underlying ZNF750 gene suppresses ESCC, PCR array was performed in ZNF750 knockdown cells, ZNF750 overexpression cells and their control cells using the kit of Cancer PathwayFinder PCR Array, which consisted of 84 genes representative of 8 different biological pathways involved in transformation and tumorigenesis, including angiogenesis, apoptosis, cell cycle, cell senescence, DNA damage and repair, EMT, hypoxia, metabolism, telomeres and telomerase.

**Figure 5:**
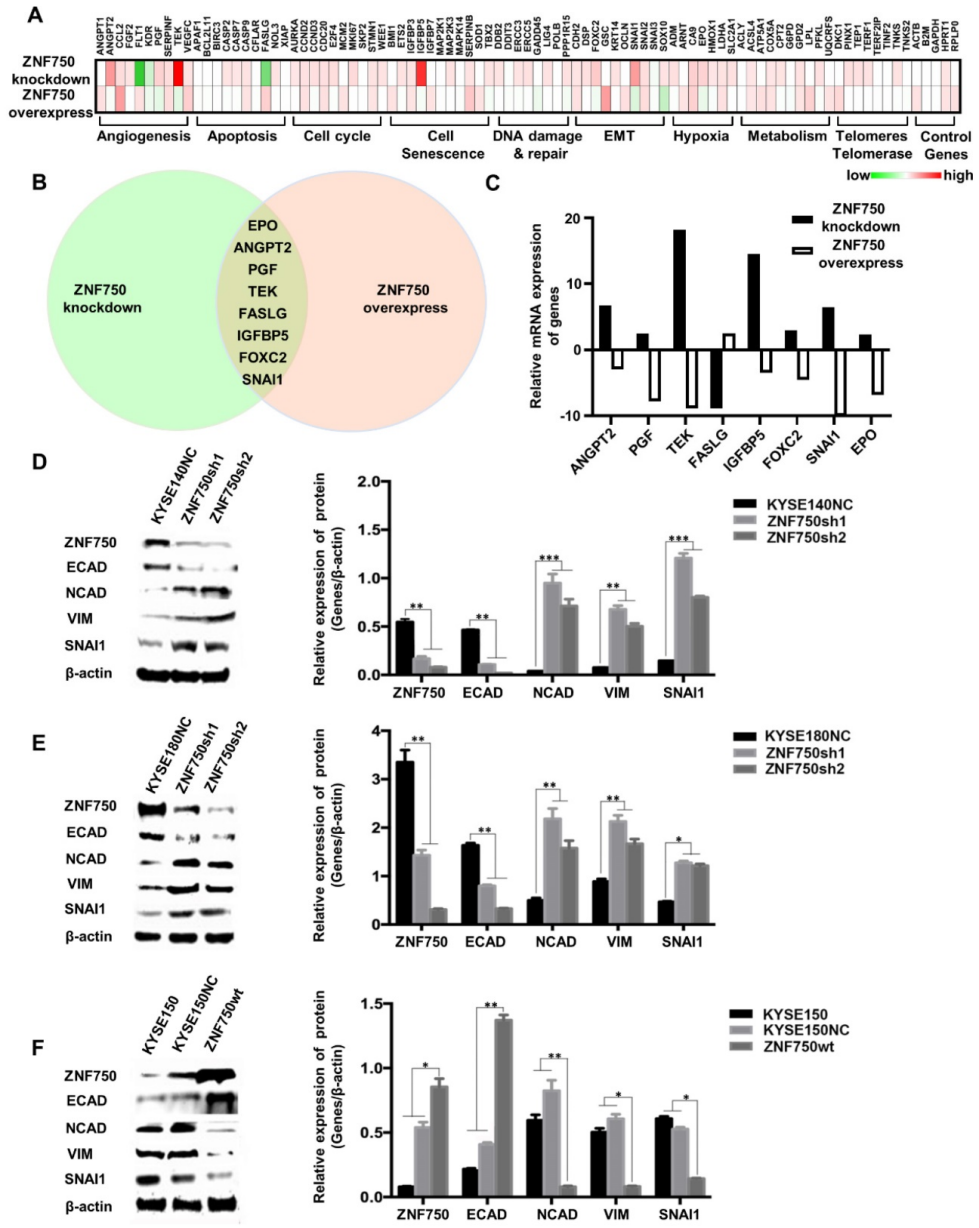
** ZNF750 inhibits the EMT process in ESCC. (A)** PCR array was performed to find the genes affected by in ZNF750 using the kit of Cancer PathwayFinder PCR Array. The green-white-red scale represents the fold-change and the fold regulation value of genes. Green: Fold down-regulation (fold change < 1); White: Fold change = 1; Red: Fold up-regulation (fold change > 1). **(B)** The Venn diagram showed EPO, ANGPT2, PGF, TEK, FASLG, IGFBP5, FOXC2, SNAI1 were the significantly changed genes in ZNF750 knockdown cells and ZNF750 overexpression cells. **(C)** Fold-regulation values of the 8 genes in ZNF750 knockdown cells and ZNF750 overexpression cells. **(D)** Decreased ZNF750 promoted the EMT process in KYSE140 ESCC cell line. **(E)** Decreased ZNF750 promoted the EMT process in KYSE180 ESCC cell line. **(F)** Overexpressed ZNF750 inhibited the EMT process in KYSE150 ESCC cell line. The protein levels of ZNF750, ECAD, NCAD, Vimentin and SNAI1 were detected by Western blot. β-actin was used as a loading control. (* *P* < 0.05, ** *P* < 0.01, *** P < 0.001).

The heatmap of PCR-array showed that angiogenesis, apoptosis and EMT were the main pathways affected by ZNF750 (Figure 5A). The Venn diagram showed Erythropoietin (EPO), Angiopoietin 2 (ANGPT2), Placental Growth Factor (PGF), TEK Receptor Tyrosine Kinase (TEK), Fas Ligand (FASLG), Insulin-like Growth Factor Binding Protein 5 (IGFBP5), Forkhead Box C2 (FOXC2), SNAI1 were the significantly changed genes, which had fold-change values greater than 2 or less than 0.5 (fold-regulation values less than -2) and have reverse trends in ZNF750 knockdown cells and ZNF750 overexpression cells (Figure 5B-C). Considering that ZNF750 inhibited the abilities of proliferation, colony formation, migration and invasion of ESCC cells, we focused on the EMT pathway and SNAI1, which was the gene with the most significant change in EMT pathway. To validate whether EMT might be a significant process affected by ZNF750, we measured the expression of EMT markers and SNAI1 when ZNF750 was knocked down or overexpressed. The results showed when ZNF750 was knocked down, the protein expression of Cadherin 1/E-cadherin (CDH1/ECAD) was significantly decreased and the expression of Cadherin 2/N-cadherin (CDH2/NCAD), Vimentin (VIM) and SNAI1 was increased (Figure 5D-E). When wild-type ZNF750 was overexpressed, the expression of the markers was changed conversely (Figure 5F). These results indicated that EMT process might be inhibited by ZNF750, and SNAI1 might be one of the downstream targets of ZNF750.

### ZNF750 directly binds to the promoter of SNAI1 and inhibits its transcription in ESCC cells

**Figure 6:**
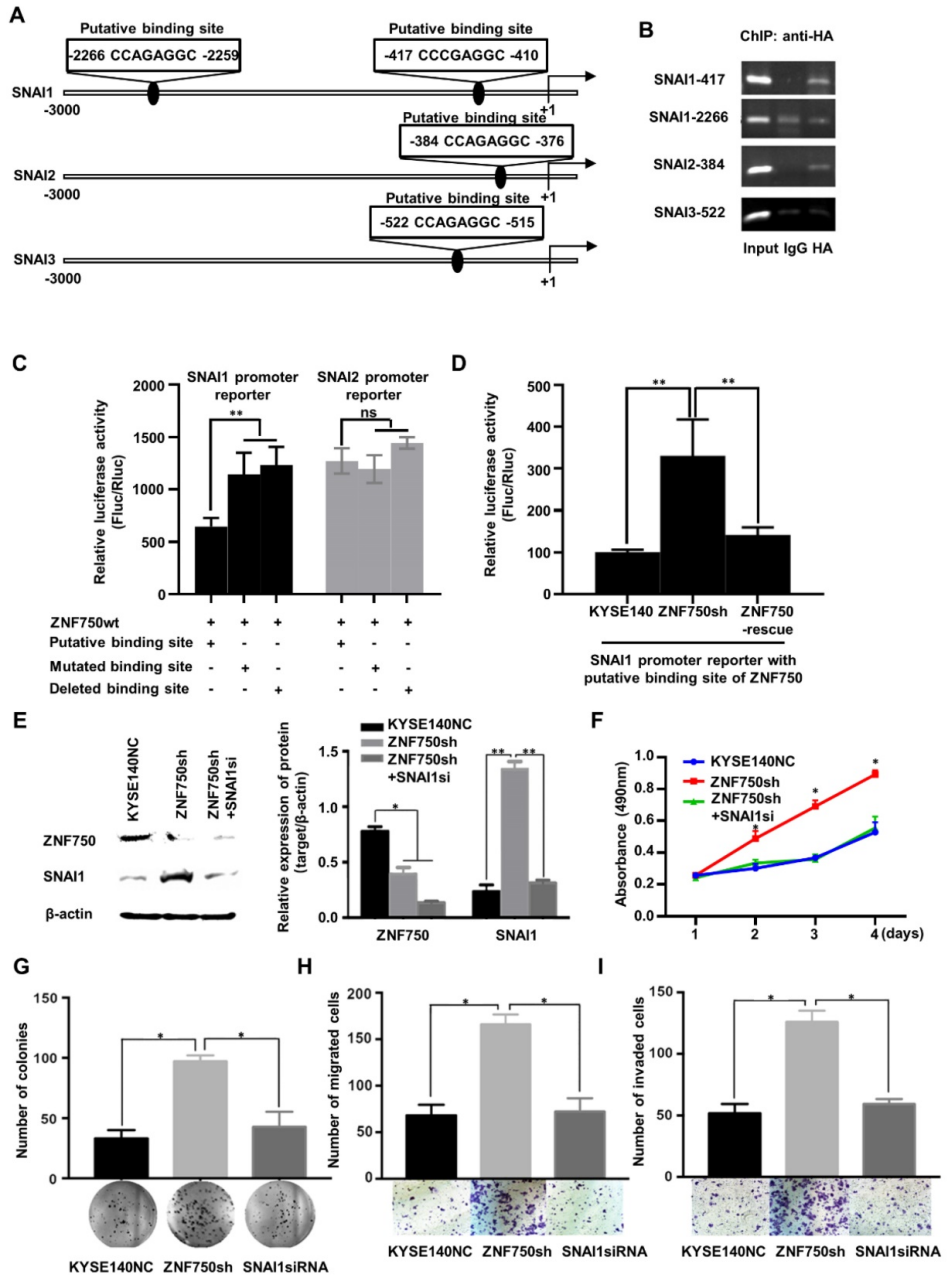
** ZNF750 binds to the promoter of SNAI1 and inhibits its activity in ESCC cells. (A)** The putative binding sites in the promoter regions of SNAI1, SNAI2 and SNAI3. **(B)** ChIP showed ZNF750 binds to the promoter of SNAI1 and SNAI2 in ESCC cells. **(C)** ZNF750 inhibited the promoter activity of SNAI1 through directly binding to the promoter region from -417 to -410 but didn't affect the promoter activity of SNAI2. **(D)** ZNF750 rescue significantly decreased the reporter activity of pGL3-SNAI1-promoter (-417 to -410) induced by ZNF750 knockdown. **(E)** SNAI1 knockdown in KYSE140 cell line with ZNF750 knockdown; The protein levels of ZNF750 and SNAI1 were detected by Western blot. β-actin was used as a loading control. **(F)** SNAI1 knockdown inhibited the proliferation induced by ZNF750 knockdown. **(G)** SNAI1 knockdown inhibited the colony formation induced by ZNF750 knockdown. **(H)** SNAI1 knockdown inhibited the migration induced by ZNF750 knockdown. **(I)** SNAI1 knockdown inhibited the invasion induced by ZNF750 knockdown. * *P* < 0.05, ** *P* < 0.01, *** P < 0.001, ns: Non-significant.

As a transcription factor, ZNF750 binds to the special site CCNNAGGC on the target genes and activates or suppresses target genes' transcription 11. To explore the molecular mechanism underlying ZNF750 regulated the expression of genes involved in EMT, we screened the promoter sequence of SNAI1, and another two Snail Family members, SNAI2 and SNAI3, to search the special binding site of ZNF750. We found two putative binding sites of ZNF750 in the promoter region of SNAI1 from -417 to -410 and from -2266 to -2259 relative to the transcription start site (TSS). Similarly, there were two putative binding sites of ZNF750 in the promoter region of SNAI2 from -384 to -376 and in the promoter region of SNAI3 from -522 to -515, respectively (Figure 6A). Further chromatin immunoprecipitation (ChIP) assay showed that ZNF750 bound to the DNA fragment from -417 to -410 in SNAI1 promoter, however, it couldn't bind to the DNA fragment from -2266 to -2259 (Figure 6B). In addition, ZNF750 could bind to the DNA fragment from -384 to -376 in SNAI2 promoter. To further assess the regulatory activity of ZNF750 on the SNAI1 and SNAI2 promoter, we performed a dual luciferase assay. The results showed when ZNF750 was overexpressed, the reporter activity of pGL3-SNAI1-promoter (-417 to -410) was decreased compared with the control (Figure 6C). When the special site CCNNAGGC was deleted or mutated, the inhibitory effect was weakened. However, the promoter of SNAI2 was not affected by ZNF750 (Figure 6C). Meanwhile, ZNF750 rescue significantly decreased the reporter activity of pGL3-SNAI1-promoter (-417 to -410) induced by ZNF750 knockdown (Figure 6D). These data demonstrated that SNAI1 might be a downstream target of ZNF750, and ZNF750 may negatively regulate the promoter activity and expression of SNAI1 through directly binding to the promoter region of SNAI1 from -417 to -410.

To confirm whether ZNF750 inhibited EMT through transcriptional repression of SNAI1, we silenced SNAI1 expression in ZNF750 knockdown ESCC cells (Figure 6E) and detected the change of biological behavior of ESCC cells. The results showed SNAI1 knockdown decreased the proliferation (Figure 6F), colony formation (Figure 6G), migration (Figure 6H) and invasion (Figure 6I) induced by ZNF750 knockdown in KYSE140 cells. The results indicated that the inhibitory effect of ZNF750 on EMT may depend on its transcriptional repression of SNAI1, and SNAI1 inhibition may partially reverse the EMT process induced by ZNF750 knockdown.

### ZNF750 is negatively correlated with SNAI1 in ESCC and other SCC clinical samples

**Figure 7:**
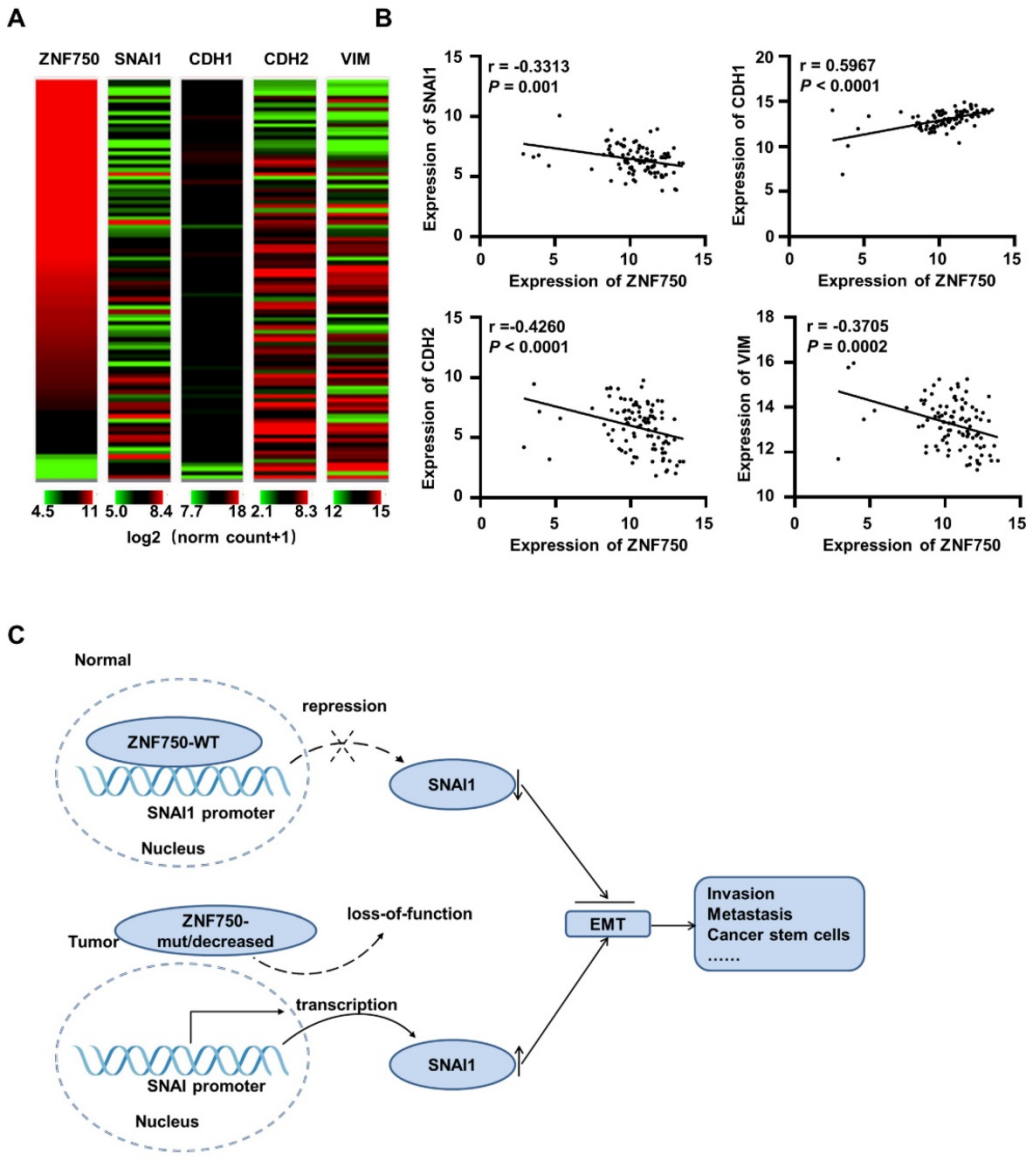
** ZNF750 is negatively correlated with SNAI1 in ESCC clinical samples based on TCGA data. (A)** The heatmap of ZNF750, SNAI1, CDH1, CDH2 and VIM expression in ESCC based on the UCSC Xena platform. Default heat map colors were in the green-black-red scale where the color green represents relatively low expression and red represents relatively high expression. Expression of the genes was based on TCGA RNA-seq data (log2 (norm_count+1)). Ranges of the five genes were shown under the columns respectively. **(B)** ZNF750 was positively correlated with ECAD/CDH1 and negatively correlated with SNAI1, CDH2 and VIM expression in ESCC. **(C)** Diagram showing how loss-function of ZNF750 contributes to tumorigenesis of ESCC via regulation of EMT process.

To further confirm the correlation ZNF750 and SNAI1 in various types of SCC, the TCGA data of esophageal cancer (ESCA, n=186 and 98 ESCC samples were included), LUSC (n=502), HNSC (n=522) and CESC (n=251) were analyzed. The results showed ZNF750 was negatively correlated with SNAI1, NCAD/CDH2 and VIM, and positively correlated with ECAD/CDH1 in ESCC (Figure 7A). Based on TCGA data of ESCA (n = 98), ZNF750 was positively correlated with ECAD (*r* = 0.5967, *P* < 0.0001) and negatively correlated with SNAI1 (*r* = -0.3313, *P* = 0.001), NCAD (*r* = -0.4260, *P* < 0.0001) and VIM (*r* = -0.3705, *P* = 0.0002) (Figure 7B). The correlations of ZNF750 with SNAI1, VIM, CDH2 and CDH1 were verified with our RNA sequencing data of 155 paired of ESCC and matched non-tumor tissues (Figure S6A and S6B). Similarly, ZNF750 was positively correlated with ECAD and negatively correlated with SNAI1, NCAD and VIM in LUSC (Figure S7A-B), HNSC (Figure S7C- D), and CESC (Figure S7E-F). These results suggested that the negative regulation of ZNF750 on SNAI1 might be a universal mechanism in SCC.

Thus, we suppose that when ZNF750 is wild type and expressed highly, it inhibits EMT process by directly binding to the promoter of SNAI1 and depressing its expression. When ZNF750 is mutated or decreased, its inhibitory effect is attenuated, and SNAI1 is activated and promotes the EMT process and malignant progression of ESCC. Further, the mechanism might be universal in various types of SCC (Figure 7C).

## Discussion

In this study, we revealed the tumor suppressive role of ZNF750 gene in ESCC and explored its underlying mechanism. We found ZNF750 was decreased in ESCC tumors compared to that of paired non-tumor tissues and the ratio of ZNF750 protein in nucleus and cytoplasm may be a promising prognostic biomarker for ESCC patients. ZNF750 bound to the promoter region of SNAI1, an essential regulator of EMT, and suppressed SNAI1 expression, thus inhibited EMT process in ESCC. The TCGA data further confirmed the negative correlation between ZNF750 and SNAI1 in ESCC and other types of SCC.

Previous study has shown ZNF750 has a relatively higher expression in esophagus than in other organs 15. Genome sequencing data had revealed that there were a large number of inactivating mutations of ZNF750 in several ESCC cohorts from China. However, ZNF750 mutations were not found in the data from whole-genome sequencing and whole exon sequencing of EAC 20. The data from TCGA also showed the inactivating mutation rate of ZNF750 in LUSC was much higher than that in the corresponding adenocarcinoma. Pan-cancer study of 21 kinds of tumors from Lawrence et al. also implicated that ZNF750 harbored many early frameshift and nonsense mutations in head and neck and lung squamous cancers 21. Therefore, ZNF750 may play a key role in the evolutionary progression of ESCC and the somatic mutations of ZNF750 may be a driving event in the occurrence and development of ESCC, even in the majority of SCC.

The role of ZNF750 in the normal squamous epithelium has been researched in skin tissues. In normal tissue, ZNF750 is necessary for the turn on the terminal epidermal differentiation gene reprogram 10, and essential for epidermal differentiation 22. ZNF750 can activate the expression of Kruppel Like Factor 4 (KLF4), a key transcription factor that activates late epidermal differentiation, via binding with the special sites on the TSS region of KLF4 gene 10. ZNF750 also activates differentiation genes or represses progenitor genes by interacting with KLF4 and chromatin regulators REST Corepressor 1 (RCOR1), Lysine Demethylase 1A (KDM1A), and C-Terminal Binding Protein 1/2 (CTBP1/2)^11^. Therefore, ZNF750 plays an important role in the differentiation of squamous epithelial cells. As known, dedifferentiation is an essential character of cancer and may be a possible cause of tumorigenesis and cancer stem cells 23. As a frequently and significantly mutated gene in ESCC, we also observed copy number loss of ZNF750 in our previous cohort 5. Thus, as a key differentiation inducer of epidermal cells, ZNF750 may be a tumor suppressor in SCC. Its truncation mutation, or copy number loss, or other factors, may cause its inactivation or down-regulation, which may lead to the dedifferentiation and reprogram of squamous epithelium cells and result in tumorigenesis of ESCC.

Our results showed ZNF750 knockdown significantly enhanced the abilities of invasion and migration of ESCC cells, and its exogenous expression significantly decreased the abilities. Moreover, PCR-array and ChIP results suggested its function was related to EMT pathway. EMT is a key process during embryogenesis and cancer progression, through which embryonic epithelial cells or epithelial cancer cells change their shape and polarity and get the ability of migration 24-26. This process is mediated by several key transcription factors, including SNAI1/2, Twist Family BHLH Transcription Factor 1 (TWIST1), Zinc Finger E-Box Binding Homeobox 1/2 (ZEB1/2) and others. In the process, the transcription factors are up-regulated and inhibit the expression of epithelial cell markers such as ECAD, keratin, and promote the expression of mesenchymal markers such as NCAD, VIM at the transcription level. Meanwhile, the epithelial cell polarity is disappeared, cell adhesion is decreased, and the abilities of migration and motility are increased, and the epithelial cells change into the cells with mesenchymal characters 24, 27-29. Evidence suggests EMT plays a critical role in tumor invasion and migration and metastasis. In ESCC, the expression of EMT-related genes, for example, SNAI1 30-32, SNAI2 33, 34, TWIST1 35, were associated with the prognosis of ESCC patients. Our results showed SNAI1 was a direct downstream target gene of ZNF750, and its expression was inhibited by ZNF750 on the transcription and protein level. Therefore, the decrease or inactivation of ZNF750 may lead SNAI1 to be free from the expression inhibition and trigger the EMT process associated with increased cancer invasion and metastasis. The mechanism may be universal in various types of SCC and indicate the important role of ZNF750 in ESCC and other types of SCC.

The studies on ESCC patients from Japan also showed ZNF750 expression predicted sensitivity to chemoradiotherapy and its mRNA level might be a promising biomarker of poor outcomes in ESCC 36. Our present results showed ZNF750 protein located in nucleus and cytoplasm, and the ratio of ZNF750 protein in nucleus and cytoplasm might be a promising prognostic biomarker for ESCC patients. An enlarger cohort and further research are needed to validate the clinical value of ZNF750.

In conclusion, our work shows that ZNF750, a novel significantly mutated gene in ESCC, may act as a tumor suppressor via directly regulating SNAI1 expression and inhibit the EMT process of ESCC cells. Its decrease or inactivation may result in tumorigenesis and progression of ESCC, even in other types of SCC. These findings are helpful to understand the potential mechanisms that drive the development of ESCC and may provide a potential strategy to overcome the disease.

## Materials and Methods

### Clinical samples

All 308 ESCC tissues and paired non-tumor tissues were collected from patients recruited from Shanxi Cancer Hospital of Shanxi Medical University. Use of these tissues was approved by the Institutional Reviewing Board and the Research Committee of Shanxi Medical University, and written consent was obtained from all participants. The patients were without preoperative chemotherapy and other treatments. And the age ranges from 29 to 79, and the median age is 61. Tissue samples were diagnosed as ESCC using hematoxylin and eosin (H&E) staining by two pathologists independently. Clinical staging of cancer was determined according to American Joint Commission for Cancer (AJCC)/International Union Against Cancer (UICC) TNM staging system (the 7th) and 31, 171, and 106 patients present with stage I, II, and III, respectively. Survival time was defined as the time from primary surgery to death or terminal time of follow-up without any events.

### Cell Culture

ESCC cell lines KYSE140, KYSE150, KYSE180, KYSE410, KYSE450, KYSE510, Colo680N, ECA109, and immortal embryonic esophageal epithelium cell line SHEE were purchased from Cell Bank of Type Culture Collection of Chinese Academy of Sciences. And HEK-293T cell was used as a packaging cell line to produce viruses. All cells were grown in RPMI 1640 (Gibco, Sigma-Aldrich, USA) or DMEM with Nutrient Mixture F-12 (DMEM/F-12, Gibco, Sigma-Aldrich, USA) supplemented with 10% fetal bovine serum (FBS, Gibco, Sigma-Aldrich) and cultured in 5% CO_2_ at 37 °C.

### Over-expression and Knockdown of ZNF750 in ESCC Lines

pMSCV-puro-ZNF750 was a generous gift from Paul A. Khavari professor (Stanford University). The coding sequence (CDS) of ZNF750 gene was subcloned into the pcDNA3.1 vector with an HA tag and validated by sequencing and Western blot. For stable expression, the CDS of ZNF750 gene was cloned into pLV-EGFP(2A)-puro-GFP and the empty vector was used as a control. For ZNF750 knockdown, three shRNA sequences for ZNF750 were designed and cloned into the PLKO.1-puro vector. A non-specific targeting shRNA was also cloned into the pLKO.1-puro vector and acted as a scrambled control (SCR). And the viruses were packaged by GenePharma Company (Shanghai, China). The viruses were added to the target cells with an appropriate multiplicity of infection (MOI). After 72 hours (hr) or 96 hr, the cells were collected and subjected to subsequent experiments or selected with puromycin to stable cells. After 5 days of selection, exogenous expression and knockdown efficiency were detected by western blot and qPCR.

### MTT assay

Cell proliferation was assessed with MTT assay. Briefly, ESCC cells were plated at a density of 5×10^3^/well into 96-well plates and incubated in normal conditions. After 24 hr, 48 hr, 72 hr and 96 hr, cells were added with 20 μl of 5 mg/ml of MTT (Invitrogen, USA) solution and incubated for 4 hr at 37 ℃. Then the MTT solution was discarded and 150 μl of dimethyl sulfoxide (DMSO) was added to dissolve the crystals. The absorbance was measured with a Spectrophotometer at 490 nm. Each experiment consisted of five replications and repeated at least three times.

### Cell migration and invasion assays

Cell migration and invasion abilities were examined by transwell assays. Cells with treatment were collected and resuspended with serum-free medium to the concentration of 5 × 10^5^/ml. For the migration assay, 200 μl cell suspension was added into the upper embedded culture chambers without Matrigel and 600 μl medium with 10% FBS was added into the bottom culture chamber. Three repeated holes were set for each group. The cell was incubated in 5% CO_2_ at 37 °C for 24 hr. The cells on the chamber bottom were fixed with 4% paraformaldehyde and stained with crystal violet and rinsed with phosphate-buffered saline (PBS). Random five view fields were chosen to count the number of transmigrated cells for each culture well. For the invasion assays, the upper chambers were precoated with 50 μl of Matrigel (1: 6 mixed with FBS-free media; BD Biosciences, Heidelberg, Germany) and proceeded the same as described above. Each experiment consisted of three replications and repeated at least three times.

### Colony-Formation assay

Cells were seeded at in 6-well plates at a density of 500 cells per well and incubated at 37 °C and 5% CO_2_ for 15 days. Then cells were fixed with 4% polyformaldehyde for 15 min and stained with 1% crystal violet and the numbers of colonies containing more than 50 cells were counted microscopically. The experiment was performed in triplicate and repeated three times at least.

### Mouse Xenograft assay

To determine the effects of ZNF750 on tumorigenesis and growth in vivo, we used a mouse xenograft assay with 12-14weeks old BALB/c nude female mice (total 18). 2 × 10^6^ cell KYSE150 cells with stable overexpression of ZNF750 and control vector (NC) were injected subcutaneously into nude mice. The size of xenograft tumors was measured at 7, 14, 21and 28 days respectively. After 28 days when mice were put to death under anesthesia, tumors were removed and weighed and stored at -80 ℃ for the next experiments. Tumor size was measured and presented as mean±Standard Deviation (SD). The tumor volume calculations were obtained using the formula V = (W^2^ × L)/2^37^. V is tumor volume, W is tumor width and L is tumor length. For animal studies, approval was obtained from the appropriate animal care committee of Shanxi Medical University.

### Western blot analysis

Protein levels of the genes were detected through western blot. Briefly, homogenized fresh tissues and cells were lysed in RIPA buffer (1% Triton X-100, 50 mM Tris-HCl, pH 7.6, 150 mM NaCl, 1% sodium deoxycholate, and 0.1% SDS) supplemented with cOmplete Protease Inhibitor Cocktail (Roche, Switzerland) for 30 min at 4 °C. The lysates were cleared by centrifugation at 12,000 g at 4 °C for 15 min, and protein concentrations were determined by the Bradford method. 50 μg of total protein were loaded and separated by SDS-PAGE (10% separating gel and 5% stacking gel) and transferred onto polyvinylidene fluoride (PVDF) membranes (Millipore, USA). The membranes were blocked with 5% non-fat milk for 2 hr at room temperature, then incubated with the special primary antibodies at 4 °C overnight. Followed by incubation with the horseradish-peroxidase (HRP)-labeled second antibodies, proteins were detected with a chemiluminescence reagent (TransGen Biotech, Beijing, China). Relative amount of gene product was normalized to β-actin levels. The experiments were conducted at least three times. The antibodies used in this experiment are shown as follows: ZNF750 (Santa Cruz, Dallas, TX, USA), ECAD (Abcam, Cambridge, UK), NCAD (Abcam, Cambridge, UK), VIM (Abcam, Cambridge, UK), SNAI1 (Abcam, Cambridge, UK), β-actin (Proteintech, Rosemont, IL, USA). The experiment was repeated three times at least.

### Real-time quantitative PCR (qPCR)

qPCR was used for measuring mRNA levels of ZNF750 in ESCC cell lines. Total RNA was extracted from cells using the RNA extraction reagent (RNAiso Plus, Takara, Bio Inc, Japan). qPCR was performed using the SYBR Green Premix Ex Taq^TM^ (Takara Bio Inc, Japan) following the instruction. All qPCR reactions were performed in triplicate with an Applied Biosystems StepOnePlus (ABI, Foster City, CA, USA). The relative expression of genes was determined by normalization to GAPDH expression according to the manufacturer's instructions. All qPCR experiments included a no template control and were done in triplicate and repeated three times at least. The primers for ZNF750 were as following: 5′-CAGAGCAAAGCCTCACAGCCTT-3′ (Forward) and 5′-AGGAGGGTCTCCGTTCACAACA-3′ (Reverse). The primers for GAPDH were as following: 5′-CCAGAACATCATCCCTGCCT-3′ (Forward) and 5′-CCTGCTTCACCACCTTCTTG-3′(Reverse). Quantification of the target genes expression was calculated using the 2^-ΔΔCt^ formula.

### Immunohistochemistry (IHC)

TMAs and the formalin-fixed paraffin-embedded xenograft tumor tissues were immunohistochemically stained. Briefly, sections were dewaxed in xylene, hydrated ethanol by concentration levels, then incubated with 3% H_2_O_2_ at room temperature for l0min to block endogenous peroxidase activity. After antigen retrieval and non-specific antigen blocking, sections were incubated with the first antibody (ZNF750 (Santa Cruz, Dallas, TX, USA), Ki-67(Abcam, Cambridge, UK)) for overnight at 4 °C; then add horseradish peroxidase-conjugated secondary antibody and incubated 30-40 min. DAB plus kit (MaiXin, Fuzhou, China) was used to develop the staining. Counterstaining was done with hematoxylin, and the sections were dehydrated in alcohol before mounting. Images were captured at 20×. The expression of ZNF750 and Ki-67 was determined using a fully automatic digital pathological scanning apparatus (Aperio, Vista, CA, USA) and analyzed using Image Scope software v12.0 (Aperio, Vista, CA, USA).

### PCR array

To explore the genes affected by ZNF750, PCR array was performed according to the manual using the Cancer PathwayFinder PCR Array kit (Qiagen, Hilden, Germany). In brief, total RNA was isolated by Trizol (Invitrogen, Waltham, MA, USA) and used to reverse transcription with an RT-PCR Kit (PrimeScript RT Master Mix, Takara, Shiga, Japan). For reverse transcription, 1 μg of total RNA was mixed with 10 μl of RT Master Mix and the total volume was 20 μl. Reverse transcription was performed at 42 °C for 15 minutes, followed by an inactivation reaction at 95 °C for 5 min. The first-strand cDNA was added to RT2 SYBR Green Master mix and aliquot the mix into RT2 Profiler PCR Arrays. PCR arrays were performed on ABI 7500 (ABI, USA). The real-time PCR program consisted of an initial denaturation at 95 °C for 10 min followed by 40 cycles of denaturation at 95 °C for 15 s and annealing at 60 °C for 1 min. The housekeeping gene GAPDH was used to normalize the amount of RNA. Quantification of the target genes expression was calculated using the 2^-ΔΔCt^ formula.

### Immunofluorescence

HA-ZNF750wt plasmid and empty vector were transfected into KYSE140 cells respectively. Then the cells were 4% formaldehyde fixed for 10 min, permeabilized with 0.1% Triton X-100 for 5 min and then incubated in 1%BSA for 1 hr to block non-specific protein-protein interactions. The cells were incubated with rabbit anti-HA antibody (Abcam, Cambridge, UK, 5 µg/ml) overnight at 4 °C, followed by Alexa Fluor® 594 goat anti-rabbit IgG antibody (Thermofisher, Carlsbad, USA, 1:1000) for 30 min and washed three times in PBS. DAPI was used to stain cell nuclei at a concentration of 0.5 µg/ml. Images were acquired by the Olympus IX71 imaging system (×200).

### Chromatin immunoprecipitation assay

The chromatin immunoprecipitation (ChIP) assay was performed using a ChIP assay kit (Millipore, Burlington, MA, USA) according to the manufacturer's instructions. HA-tagged FOXF2 plasmid was transfected into KYSE140 cells, anti-HA antibody (Abcam, Cambridge, UK) was used to enrich the DNA fragments containing the putative ZNF750 binding site of target genes and the isotype IgG (Abcam, Cambridge, UK) was used as a negative control. The sequences for primers used for the amplification of SNAI1, SNAI2 or SNAI3 promoter regions containing a putative ZNF750 binding site were as follows: 5′-CAGTGATGTGCGTTTCCCTC-3′ and 5′-GTGGTCTGAGCGCTTCTG-3′ for SNAI1 promoter region (from -417 to -410), 5′- GGGTTTGGGCTGGACAAC-3′ and GCAGCACATTAGATCCCCAC-3′ for another SNAI1 promoter region (-2266 to -2259), 5′-ACCTCTCCAGATGCCACTTC-3′ and 5′-GGTTCAAAATGGGCTGTTTT-3′ for SNAI2 promoter (-384 to -376), 5′-ACGGAAGACACGGAAAGGAT-3′ and 5′-TGCAGACTCACTCTTCAGCT-3′ for SNAI3 promoter (-522 to -515).

### Dual-Luciferase Reporter Assay

The reporter plasmids of the promoter of SNAI1 were constructed according to the sequence of the SNAI1 promoter from the UCSC Genome Browser website. The SNAI1 promoter region with (-417 to +1) or without (-410 to +1) ZNF750 binding site or mutated site was amplified from human genomic DNA using the primers 5′-GCAACGCGTTTTCCGCCCCCTCCCAAG-3′ (forward) or 5′-GCAACGCGTGCGGGCGTCGGAAGGTCAGGT-3′ (forward) or 5′-GCAACGCGTTAAGACTGTGTCGGGGGCGGGCGT-3′ (forward) and 5′-GCAAGATCTTAGTGGTCGAGGCACTGGGGT-3′ (reverse) with MluI and Bgl II restriction endonuclease recognition sites at the 5′-ends, respectively. The DNA fragments were cloned into pGL3-Basic (Promega, Madison, WI, USA). The pGL3 reporter and pRL-TK plasmid were transiently co-transfected into KYSE150 and KYSE140 cells for 48 hr. The luciferase activity of pGL3-SNAI1 (-417 to +1) or pGL3-SNAI1Δ(-410 to +1) or pGL3-SNAI1-mut was normalized to Renilla luciferase activity. The assay was performed according to the instruction. The experiment was performed in triplicate and repeated three times at least.

### Bioinformatics and data analysis

Somatic mutation profiles of ZNF750 gene in ESCC were obtained from our previous reports and the reports by Lin et al. 6, Song et al.^7^, Gao et al.^18^. Somatic mutation and copy number data of ZNF750 in varied cancer types, including ESCC, EAC, LUSC, LUAC, HNSC, CESC were collected from TCGA via cBioPortal for Cancer Genomics (https://www.cbioportal.org/)^38^, ^39^ and analyzed by its oncoprint function. Expression data of various genes, including ZNF750, SNAI1, ECAD (CDH1), NCAD (CDH2) and VIM in ESCC, LUSC, HNSC, and CESC were collected from TCGA via xenabrowser (https://xenabrowser.net/heatmap/)^40^ and analyzed by Graphpad Prism software (GraphPad Software, La Jolla, CA, USA). RNA-sequencing data of 155 paired of ESCC cohort was from our another study.

### Statistical Analysis

Data were presented as mean ± SD or mean ±SEM. The means were compared using Student's t-test, and data from more than two groups were analyzed by one-way ANOVA. The correlation between ZNF750 expression and the clinical variables in ESCC was analyzed by Chi-square test. Survival analysis was carried out according to Kaplan-Meier analysis and Log rank test or Breslow test. Multivariate survival analyses were performed by a Cox proportional hazards regression model. All statistical analyses were performed using Statistical Package for Social Science for Windows (SPSS, version 20.0; IBM Inc, USA). The correlations between genes were performed using nonparametric correlation (Spearman) by GraphPad prism software.* P*< 0.05 was considered statistically significant.

## Supplementary Material

Supplementary figures and table.

## Figures and Tables

**Table 1:** Association between ZNF750 protein levels in primary ESCC tissues and clinicopathological variables.

Clinic features	ZNF750_low_	ZNF750_high_	*P*-value
**Age**			
** <60**	43(32.8%)	88(67.2%)	0.098
** ≥60**	75(42.4%)	102(57.6%)	
**Gender**			
** female**	39(38.2%)	63(61.8%)	1.000
** male**	79(38.3%)	127(61.7%)	
**Location**			
** upper**	36(39.1%)	56(60.9%)	0.981
** middle**	76(38.0%)	124(62.0%)	
** lower**	6(37.5%)	10(62.5%)	
**Smoking status**			
** never**	57(37.7%)	94(62.3%)	0.907
** yes**	61(38.9%)	96(61.1%)	
**Drinking status**			
** never**	83(39.9%)	125(60.1%)	0.453
** yes**	35(35.0%)	65(65.0%)	
**Grade**			
** 1**	11(40.7%)	16(59.3%)	0.901
** 2**	85(38.6%)	135(61.4%)	
** 3**	22(36.1%)	39(63.9%)	
**T stage**			
** 1+2**	50(32.9%)	102(67.1%)	0.061
** 3+4**	68(43.6%)	88(56.4%)	
**LN stage**			
** N0-N1**	99(37.9%)	162(62.1%)	0.747
** N2-N3**	19(40.4%)	28(59.6%)	
**TNM stage**			
** Ⅰ+Ⅱ**	78(38.6%)	124(61.4%)	0.902
** Ⅲ+Ⅳ**	40(37.7%)	66(62.3%)	
**Vital status**			
** Deceased**	60(45.8%)	71(54.2%)	**0.024**
** Living**	58(32.8%)	119(67.2%)	

## Abbreviations

ESCC: esophageal squamous cell carcinoma
EAC: esophageal adenocarcinoma
LUSC: lung squamous cell carcinoma
LUAC: lung adenocarcinoma
HNSC: head and neck squamous cell carcinoma
CECC: cervical squamous cell carcinoma
CNV: copy number variation
ZNF750: Zinc Finger Protein 750
NLS: nuclear localization signal
qPCR: quantitative real-time PCR
RNA: ribonucleic acid
ECAD: E-cadherin
CDH1: cadherin 1
siRNA: small interference RNA
shRNA: short hairpin RNA
EMT: epithelial-mesenchymal transition
IHC: immunohistochemistry
TMA: tissue microarray
ChIP: chromatin immunoprecipitation
NC: negative control
NCAD: N-cadherin
SNAI1: snail family transcriptional repressor 1
VIM: Vimentin
Luc: luciferase
WT: wildtype
TCGA: the Cancer Genome Atlas
UCSC: University of California, Santa Cruz
TWIST1: Twist Family BHLH Transcription Factor 1
ZEB1/2: Zinc Finger E-Box Binding Homeobox 1/2
KLF4: Kruppel Like Factor 4
RCOR1: REST Corepressor 1
KDM1A: Lysine Demethylase 1A
CTBP1/2: C-Terminal Binding Protein 1/2
HR: Hazard Ratio
SD: Standard Deviation
SE: Standard error
CI: confidence interval
