# Supplementary material for: Novel ESCC-related gene ZNF750 as potential Prognostic biomarker and inhibits Epithelial-Mesenchymal Transition through directly depressing SNAI1 promoter in ESCC
Source: Theranostics. 2020 Jan 1;10(4):1798–813. doi: 10.7150/thno.38210 (PMC6993233; doi:10.7150/thno.38210)
Supplement: Supplementary file 1 — Supplementary figures and table. [file thnov10p1798s1.pdf]

Supplementary File

Table S1. Statistical difference of survival between the four groups divided by ZNF750 combined with N stage

Group	$ZNF750_{high}$+N(0-1)		$ZNF750_{low}$+N(0-1)		$ZNF750_{high}$+N(2-3)		$ZNF750_{low}$+N(2-3)	
	χ^2	***P***	χ^2	***P***	χ^2	***P***	χ^2	***P***
$ZNF750_{high}$+N(0-1)			4.835	0.028	20.484	6.014E-6	27.953	1.243E-7
$ZNF750_{low}$+N(0-1)	4.835	0.028			7.148	0.008	11.776	0.001
$ZNF750_{high}$+N(2-3)	20.484	6.014E-6	7.148	0.008			1.324	0.250
$ZNF750_{low}$+N(2-3)	27.953	1.243E-7	11.776	0.001	1.324	0.250		

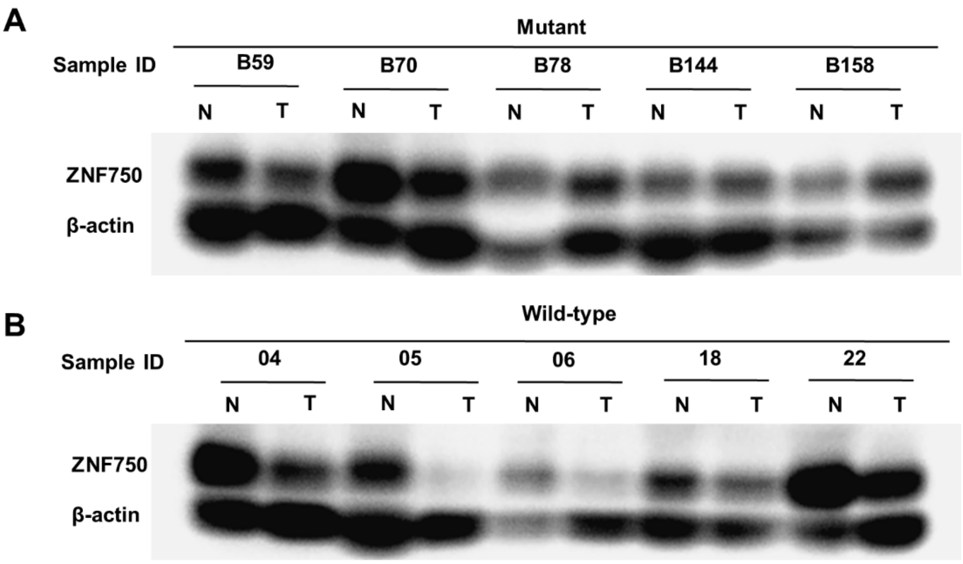

Figure S1. ZNF750 protein levels in ESCC tissues with or without mutant ZNF750. (A) ZNF750 protein levels in ESCC tissues with mutant ZNF750 and in the matched non-tumor tissues. **(B)** ZNF750 protein levels in ESCC tissues with wild-type ZNF750. N: matched non-tumor tissues; T: ESCC tissues.

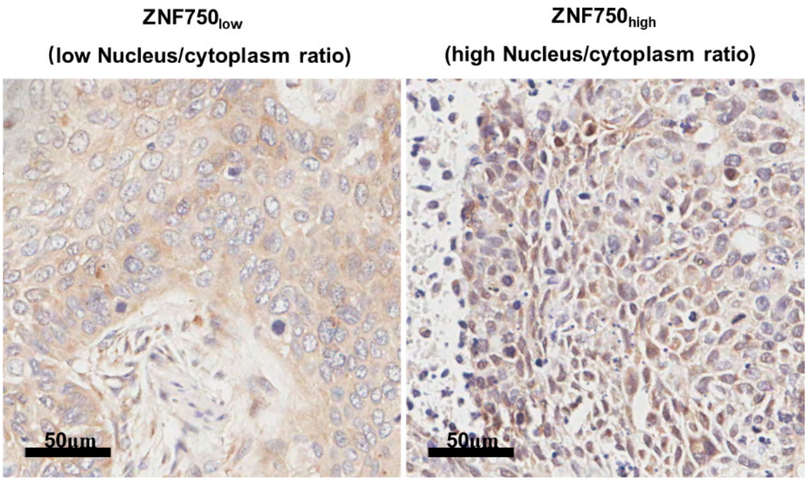

Figure S2. Representative IHC images of ESCC tissues with different ZNF750 nucleus/cytoplasm ratio. ESCC tissues were stained by rabbit anti-ZNF750 antibody and counterstained by hematoxylin. ZNF750 was stained with brown and nuclei were stained with blue. In $ZNF750_{low}$ group, ZNF750 was located in cytoplasm mainly. In $ZNF750_{high}$ group, ZNF750 was located in nuclei mainly. Scale bar was 50μm.

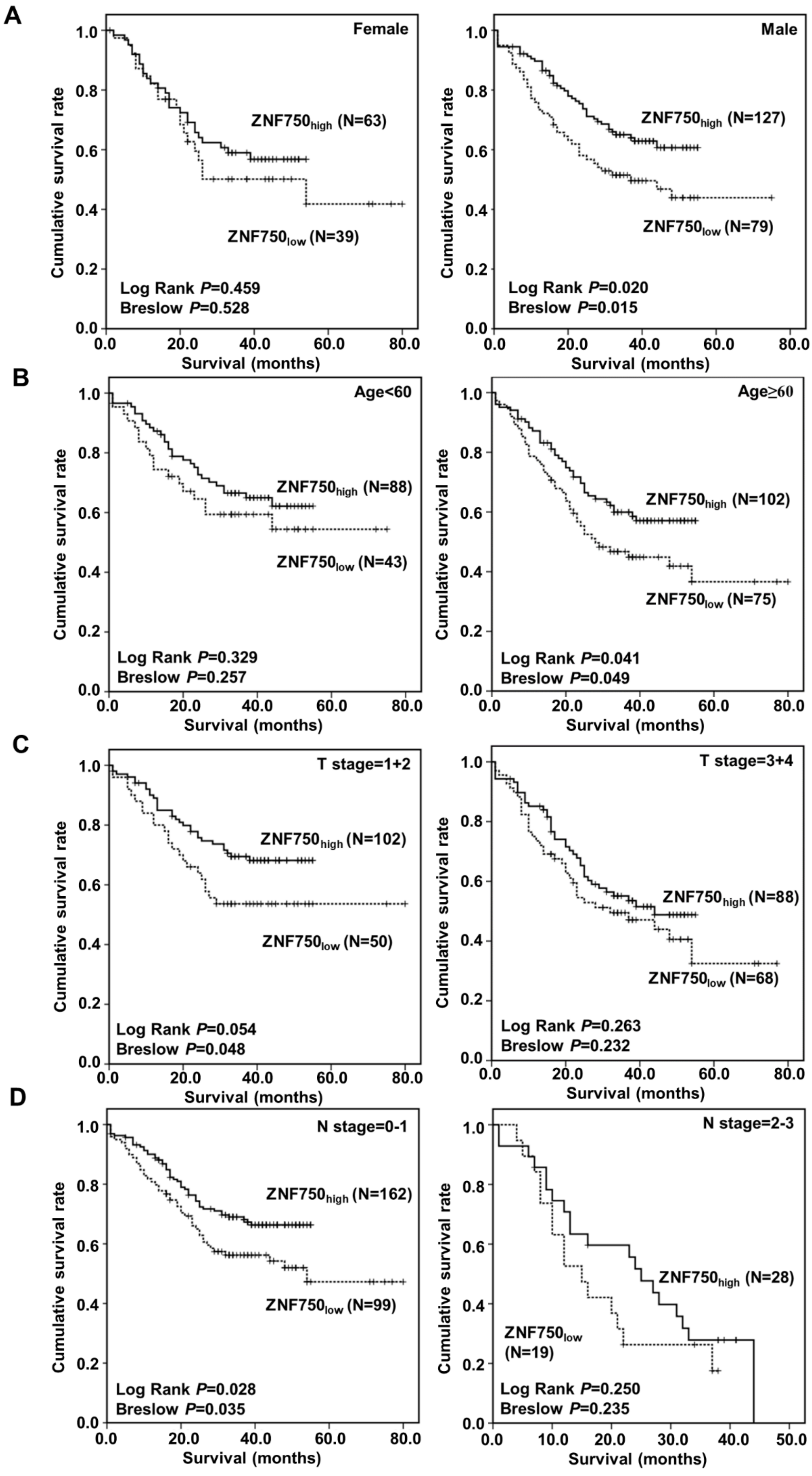
A
Female
ZNF750high (N=63)
ZNF750low (N=39)
Log Rank P=0.459
Breslow P=0.528
Male
ZNF750high (N=127)
ZNF750low (N=79)
Log Rank P=0.020
Breslow P=0.015
B
Age<60
ZNF750high (N=88)
ZNF750low (N=43)
Log Rank P=0.329
Breslow P=0.257
Age≥60
ZNF750high (N=102)
ZNF750low (N=75)
Log Rank P=0.041
Breslow P=0.049
C
T stage=1+2
ZNF750high (N=102)
ZNF750low (N=50)
Log Rank P=0.054
Breslow P=0.048
T stage=3+4
ZNF750high (N=88)
ZNF750low (N=68)
Log Rank P=0.263
Breslow P=0.232
D
N stage=0-1
ZNF750high (N=162)
ZNF750low (N=99)
Log Rank P=0.028
Breslow P=0.035
N stage=2-3
ZNF750high (N=28)
ZNF750low (N=19)
Log Rank P=0.250
Breslow P=0.235
Cumulative survival rate
Survival (months)

Figure S3. Kaplan-Meier survival plot showed the clinical value of ZNF750 nucleus/cytoplasm ratio in ESCC patients with different gender, age, T stage and N stage. (A) Kaplan-Meier survival plot showed the cumulative survival rate of ESCC patients with different ZNF750 nucleus/cytoplasm ratios in male and female groups. **(B)** Kaplan-Meier survival plot showed the cumulative survival rate of ESCC patients with different ZNF750 nucleus/cytoplasm ratios in Age<60 and Age≥60 groups. **(C)** Kaplan-Meier survival plot showed the cumulative survival rate of ESCC patients with different ZNF750 nucleus/cytoplasm ratios in T1+2 and T3+4 groups. **(D)** Kaplan-Meier survival plot showed the cumulative survival rate of ESCC patients with different ZNF750 nucleus/cytoplasm ratios in N(0-1) and N(2-3) groups. Log rank test and Breslow test were used to analyze the survival data of ESCC patients with different gender, age, T stage and N stage. And *P* values were shown in the figures. $P < 0.05$ was considered statistically significant.

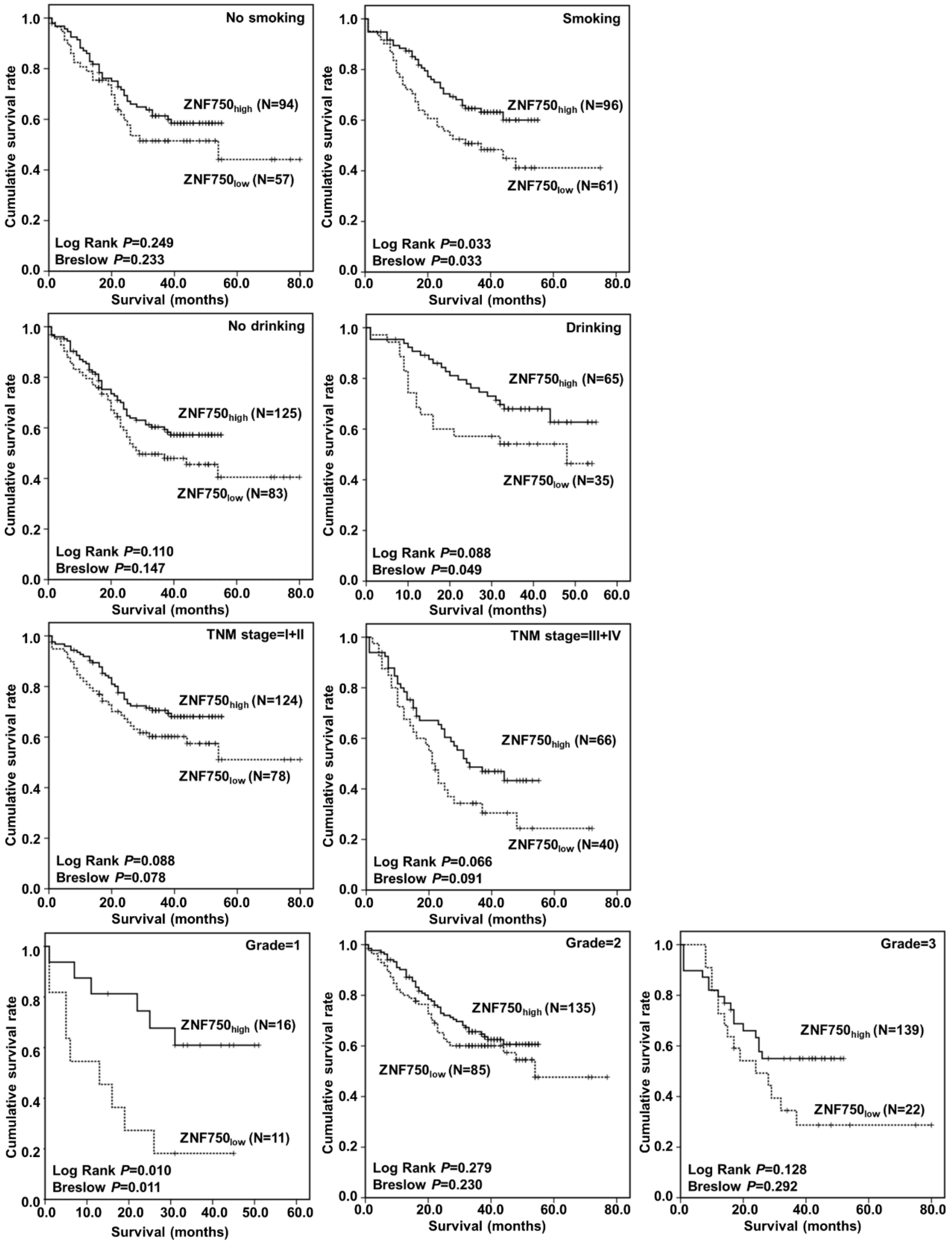

Figure S4. Kaplan–Meier survival plot showed the clinical value of ZNF750 nucleus/cytoplasm ratio in ESCC patients with different smoking status, drinking status, TNM stage and histological grade. Log rank test and Breslow test were used to analyze the survival data of ESCC patients with different smoking status,

drinking status, TNM stage and histological grade. And P values were shown in the figures. $P < 0.05$ was considered statistically significant.

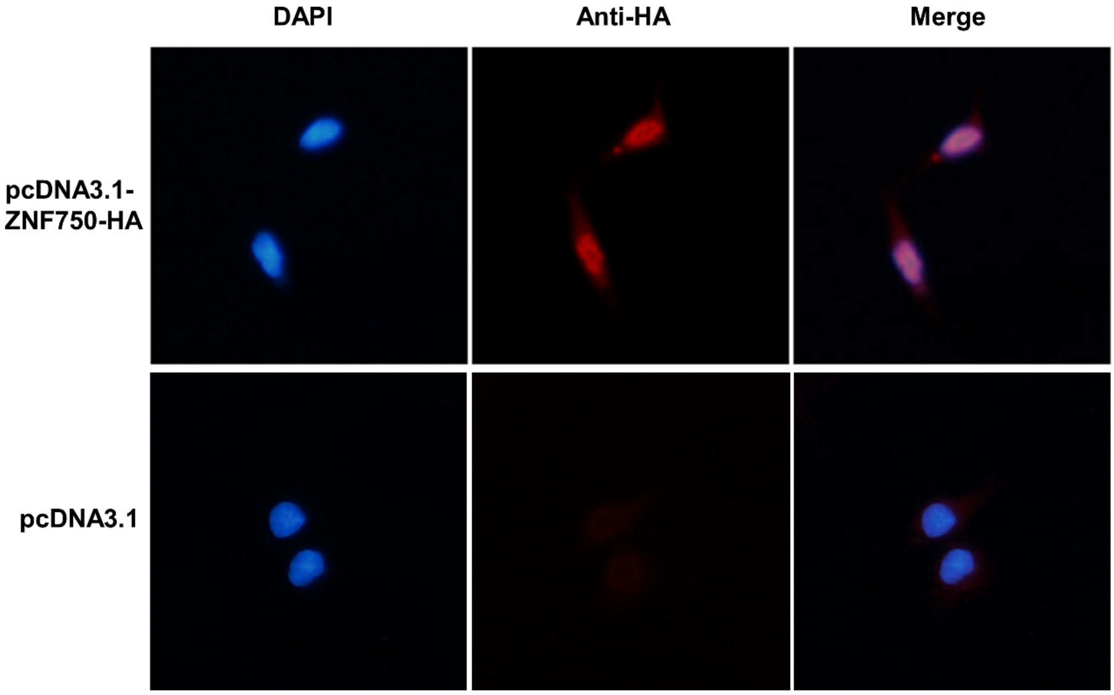

Figure S5. Immunofluorescence images of ESCC cells with HA-tagged ZNF750wt overexpression. Cells were stained with rabbit anti-HA antibody (the first antibody) and Alexa Fluor® 594 goat anti-rabbit IgG (red) acted as the second antibody. DAPI was used to stain cell nuclei (blue). The first row showed the nucleus (Blue, DAPI), overexpressed ZNF750 (red, anti-HA) and the merged image (merge). The second row showed vector control. Magnification, 200×.

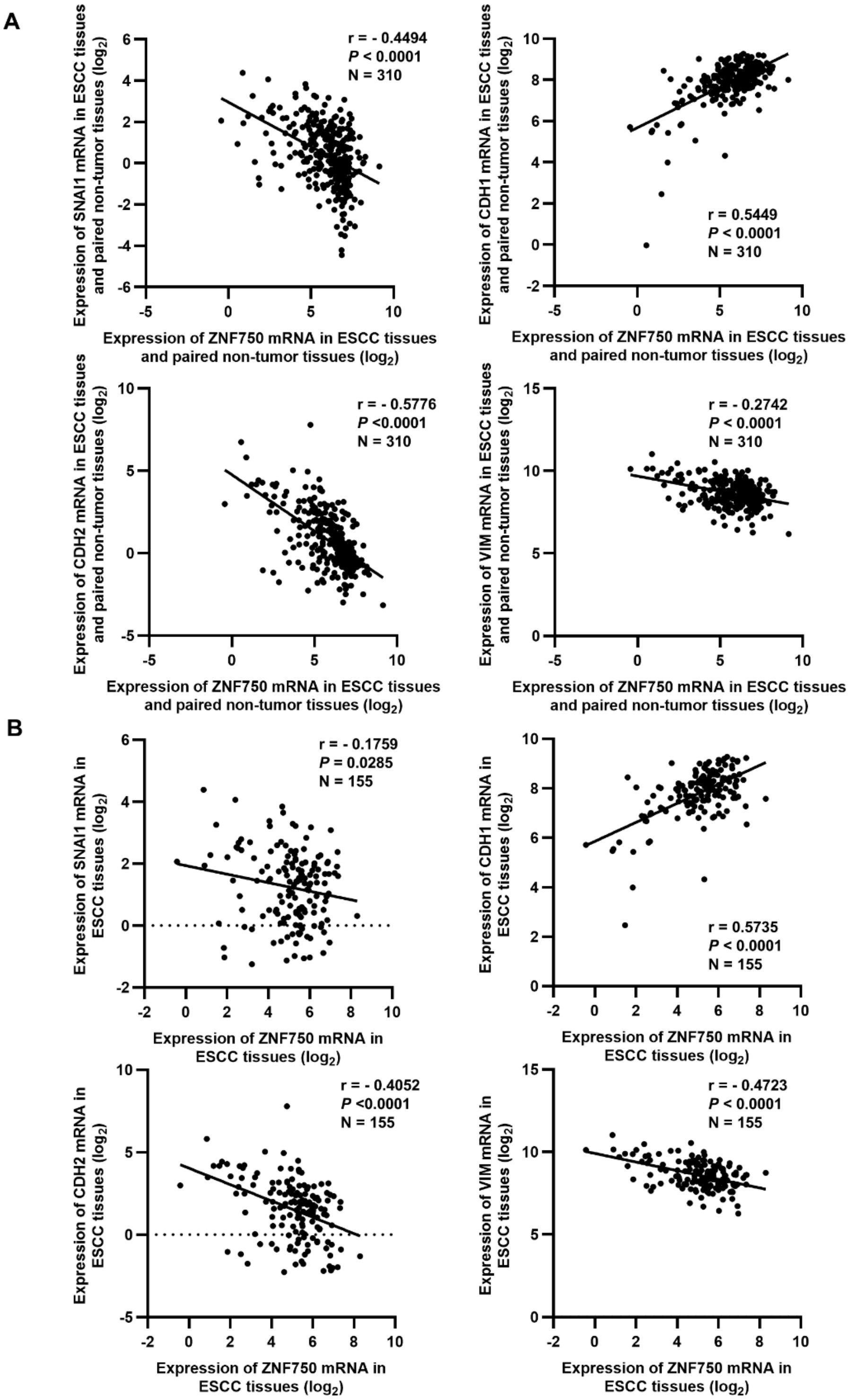

Figure S6. The correlations of ZNF750 with SNAI1, VIM, CDH2 and CDH1 were verified with RNA sequencing data of 155 paired of ESCC and matched non-tumor tissues. (A) The correlations of ZNF750 with SNAI1, VIM, CDH2 and

CDH1 in 155 paired of ESCC and matched non-tumor tissues. The nonparametric Spearman correlations were computed using GraphPad prism software. N = 310 (155 ESCC tissues plus matched non-tumor tissues). **(B)** The correlations of ZNF750 with SNAI1, VIM, CDH2 and CDH1 in 155 paired of ESCC and matched non-tumor tissues. The nonparametric Spearman correlations were computed using GraphPad prism software. N = 155 (155 ESCC tissues only). Correlation Coefficient (r) and *P* values were shown in the figures. $P < 0.05$ was considered statistically significant.

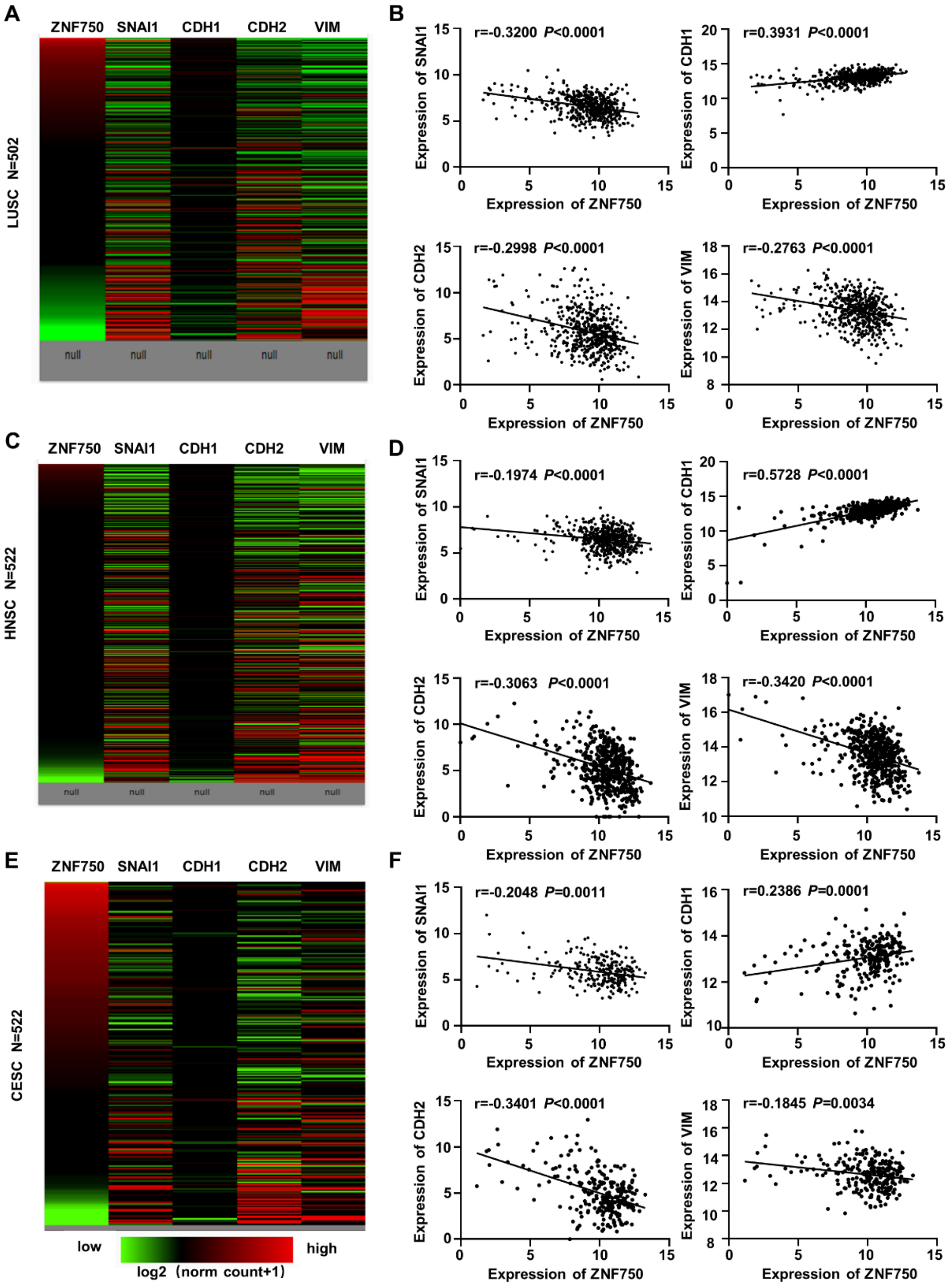

Figure S7. ZNF750 is negatively correlated with SNAI1 in different SCC clinical samples based on TCGA data. (A) The heatmap of ZNF750, SNAI1, CDH1, CDH2 and VIM expression in LUSC; the color green represents relatively low expression

and red represents relatively high expression. **(B)** ZNF750 was positively correlated with ECAD and negatively correlated with SNAI1, CDH2 and VIM expression in LUSC. **(C)** The heatmap of ZNF750, SNAI1, CDH1, CDH2 and VIM expression in HNSC. **(D)** ZNF750 was positively correlated with ECAD and negatively correlated with SNAI1, CDH2 and VIM expression in HNSC. **(E)** The heatmap of ZNF750, SNAI1, CDH1, CDH2 and VIM expression in CESC. **(F)** ZNF750 was positively correlated with ECAD and negatively correlated with SNAI1, CDH2 and VIM expression in CESC. Correlation Coefficient (r) and *P* values were shown in the figures. $P < 0.05$ was considered statistically significant.
